# Emergence of Tigecycline-Nonsusceptible Carbapenem-Resistant *Klebsiella pneumoniae* with Metallo-β-Lactamase and Transferable Ceftazidime-Avibactam Resistance in China

**DOI:** 10.3390/pathogens14030253

**Published:** 2025-03-04

**Authors:** Yajuan Ni, Jiefu Peng, Yawen Xu, Liguo Zhu, Xiao Wang, Hui Jin, Huimin Qian

**Affiliations:** 1 Department of Epidemiology and Health Statistics, School of Public Health, Southeast University, Nanjing 210009, China; yajuanni0728@163.com; 2 Key Laboratory of Environmental Medicine Engineering, Ministry of Education, School of Public Health, Southeast University, Nanjing 210009, China; 3 Department of Acute Infectious Disease Control and Prevention, Jiangsu Provincial Center for Disease Control and Prevention, Nanjing 210009, China; pengjiefu@jscdc.cn (J.P.); zhuliguo2002@163.com (L.Z.); 4 National Health Commission (NHC) Key Laboratory of Enteric Pathogenic Microbiology, Jiangsu Provincial Center for Disease Control and Prevention, Nanjing 210009, China; 5 Yangzhou Center for Disease Control and Prevention, Yangzhou 225007, China; jsyawen@163.com; 6 Xuzhou Center for Disease Control and Prevention, Xuzhou 221006, China; 15996950112@139.com

**Keywords:** carbapenem-resistant *Klebsiella pneumoniae*, tigecycline, ceftazidime-avibactam, horizontal transfer

## Abstract

In recent years, resistance of *Klebsiella pneumoniae* to the clinical last-resort drugs carbapenem and tigecycline has intensified, including Metallo-β-Lactamase-producing *K. pneumoniae* (MBL-KP), which demonstrated resistance to ceftazidime-avibactam (CZA), posing a significant public health threat. This study focused on the carbapenems, CZA, and tigecycline resistance mechanisms of MBL-producing Carbapenem-resistant *K. pneumoniae* (MBL-CRKP). A retrospective study and genomic epidemiological analysis of Carbapenem-resistant *K. pneumoniae* (CRKP) strains isolated from Yangzhou City, Jiangsu Province, China, between 2016 and 2023 was conducted. The detection rate of CRKP in Yangzhou City has increased significantly in recent years, with five strains carrying the Metallo-β-Lactamases (MBLs) gene, all of which exhibited resistance to carbapenems and CZA. Two strains even showed reduced susceptibility to tigecycline, with one harboring *tmexCD2-toprJ2*. Moreover, three CRKP strains carrying both *bla*_KPC-2_ and *bla*_NDM-1_/*bla*_NDM-29_ genes were identified. Plasmids carrying MBL genes can horizontally transfer, leading to the spread of resistance, thus further exacerbating the difficulty of clinical treatment and the spread of resistance. In conclusion, this study not only revealed the resistance of MBL-CRKP strains to clinical last-resort therapeutic drugs but also explored the resistance mechanism and horizontal transfer through genomic analysis. Moreover, this study also suggested that microbial drug resistance surveillance should be conducted from the perspective of “one health” in the future to combat this global health challenge.

## 1. Introduction

*Klebsiella pneumoniae* is a member of the *Enterobacterales* family and ranks second among the Gram-negative bacteria that cause bloodstream infections (BSI) [1]. Carbapenem-resistant *Enterobacterales* (CREs) are classified as a critical group on the World Health Organization’s Bacterial Priority Pathogen List 2024 [2]. Carbapenem-resistant *K. pneumoniae* (CRKP) is the most common CRE [3], and its prevalence of CRKP increased from 2.9% to 24.2% between 2005 and 2022 in China [4]. The global prevalence of CRKP continues to exhibit a persistent upward trend. A more alarming situation is that the proportion of CRKP infections has reached 29.7% in Italy, with infection rates exceeding 60% in Greece and Turkey [5,6,7]. The spread of CRKP and its clinical outcomes pose a considerable threat to public health [8].

Carbapenem resistance primarily arises from hydrolysis of the antibiotics by carbapenemases. The emergence of *K. pneumoniae* carbapenemases (KPCs), class D carbapenemases, OXA-48, Metallo-β-Lactamase (MBLs) such as New Delhi metallo-β-lactamase (NDM), and imipenemase (IMP) has substantially weakened the efficacy of carbapenem antibiotics [1]. Ceftazidime-avibactam (CZA) is a novel cephalosporin/β-lactamase inhibitor combination that can inhibit class A, class C, and some class D extended-spectrum β-lactamases, and it emerged as a promising treatment option for CRKP infections [9]. Avibactam irreversibly inhibits serine β-lactamases by covalently binding to their catalytic serine residue. However, MBLs lack this serine site and instead utilize Zn^2^⁺ ions to hydrolyze β-lactams, rendering avibactam ineffective [9,10]. Recently, the efficacy of CZA has been challenged by reduced antibiotic sensitivity in recently isolated CRKP strains harboring MBLs in China.

Carbapenem-resistant *K. pneumoniae* (CRKP) typically exhibits resistance to multiple antibiotics, including tigecycline, which is used as the last resort treatment option in clinical practice [11]. Recent reports have highlighted the reduced susceptibility of CRKP strains to tigecycline, which further complicates therapeutic options and poses a significant burden on human health and clinical care [12,13,14]. Tigecycline exerts its antimicrobial effect by binding to the 30S ribosomal subunit and inhibiting protein synthesis [15,16]. In *K. pneumoniae*, the tigecycline resistance mechanism is usually mediated by chromosomally encoded multi-drug resistance efflux pumps, such as AcrAB and OqxAB, which cannot horizontally transfer easily [17]. However, a new plasmid-mediated resistance nodule division (RND)-type efflux pump gene cluster, *tmexCD1-toprJ1*, has been identified in recent years. The gene cluster and its homozygous variants have been identified in animal and clinical isolates from various countries [18]. And *tmexCD2-toprJ2*, as its variant, was first identified in *Raoultella ornithinolytica* and then was found in clinical isolates across China [19]. The emergence and spread of this efflux pump gene cluster could exacerbate the bacterial drug resistance crisis. To date, no studies have reported the co-existence of metallo-β-lactamase resistance gene and *tmexCD2-toprJ2* on the same conjunctive plasmid in *K. pneumoniae* strains from the Yangzhou area, leading to resistance to carbapenems, tigecycline, and CZA [20,21,22].

Carbapenem resistance genes can spread worldwide through horizontal gene transfer (HGT) mediated by mobile genetic elements (MGEs) such as plasmids, transposons, and insertion elements (ISs) [23]. The horizontal transfer of MGEs carrying resistance genes contributes to the increasing prevalence of multidrug-resistant strains and can lead to the coexistence of multiple carbapenem-resistant genes within the same bacterial strain, posing a significant challenge for clinical treatment.

Yangzhou was selected as the study area due to its decade-long surveillance of *K. pneumoniae* antimicrobial resistance by the local CDC, reflecting regional resistance trends. In addition, the severe drug resistance in local poultry farming raised concerns about potential transmission to humans. In addition, multidrug resistance and its potential for horizontal gene transfer of Metallo-β-Lactamase-producing Carbapenem-resistant *K. pneumoniae* (MBL-CRKP) has presented considerable challenges to both clinical management and public health. Hence, this study aimed to investigate the mechanisms of resistance to carbapenems, tigecycline, and CZA of these emerging strains, which are crucial for the development of effective therapeutic strategies and the improvement of patient prognosis in Yangzhou. Meanwhile, this study further investigated the transmissibility among Chinese MBL-CRKP isolates, to inform therapeutic guidelines and public health interventions.

## 2. Materials and Methods

### 2.1. Collection and Identification of K. pneumoniae Clinical Isolates

This retrospective study analyzed 255 *K. pneumoniae* strains isolated from five major general hospitals in Yangzhou, Jiangsu Province (2016–2023). Revived strains were cultured on nutrient agar at 37 °C for 18–24 h and confirmed as *K. pneumoniae* via matrix-assisted laser desorption ionization time-of-flight mass spectrometry (MALDI-TOF-MS) (Bruker, Berlin, Germany).

### 2.2. Antimicrobial Susceptibility Testing

The minimum inhibitory concentrations (MICs) of antibiotics were determined by broth microdilution (BMD) and BD PhoenixTM M50 (BD Biotechnology Co., Ltd., Baltimore, MD, USA), with *Escherichia coli* ATCC 25922 as the quality control strain. The drug resistance phenotype was identified based on the drug breakpoints specified by the Clinical and Laboratory Standards Institute (CLSI) and the European Committee on Antimicrobial Susceptibility Testing (EUCAST). For tigecycline, CLSI currently does not provide tigecycline-specific criteria for *Enterobacterales*. The breakpoints of the US Food and Drug Administration (FDA) (susceptible, ≤2.0 µg/mL; intermediate, 4.0 µg/mL; and resistant, ≥8.0 µg/mL) were authoritative and applicable. CRKP was defined as resistance to ≥1 carbapenem. Strains not susceptible to ≥3 antibiotic classes in addition to ampicillin (to which all *K. pneumoniae* infections are intrinsically resistant) were considered multidrug-resistant (MDR) *K. pneumoniae*.

### 2.3. DNA Extraction

Genomic DNA was extracted using the FastPure^®^ Bacteria DNA Isolation Mini Kit (Vazyme, Nanjing, China). The colonies were added to a 1.5 mL centrifuge tube containing 230 µL Buffer GA and shaken until the bacteria were thoroughly suspended. A quantity of 20 µL Proteinase K was added, then 250 µL Buffer GB was added, and it was shaken and mixed well, followed by a 70 °C water bath for 10 min. A quantity of 4 µL RNase A was added to the digest, and it was shaken for 15 s and left at room temperature for 5–15 min. A quantity of 180 µL ethanol absolute was added, it was shaken and mixed well and centrifuged briefly, and then the mixture was put into the FastPure gDNAMini Columns collection tube. Quantities of 500 µL PB and 600 µL PW were added sequentially to wash out impurities according to the protocol, and finally, Elution Buffer was added to elute the DNA on the column. A Qubit 2.0 Fluorometer (Thermo, Waltham, MA, USA) was used for quantification.

### 2.4. Whole-Genome Sequencing and Nanopore Sequencing

Whole-genome sequencing was performed on an Illumina Novaseq platform (150 bp paired-end) (Illumina, San Diego, CA, USA). Furthermore, five MBL-CRKP isolates underwent additional Nanopore long-read sequencing. Hybrid assemblies were generated using CLC Genomics Workbench 23.0 (Qiagen, Hilden, Germany).

### 2.5. Real-Time Quantitative Reverse Transcription PCR and Mutation Analysis

The presence of *ramA*, *ramR*, *rarA*, *acrR*, *acrA*, *acrB*, *soxS*, *oqxA*, *oqxB*, and *tolC* within the two tigecycline-nonsusceptible CRKP strains was confirmed by BLAST+ 2.9.0. Total RNA extraction (RNeasy mini kit, Qiagen, Hilden, Germany) and quality control (NanoDrop One spectrophotometer, Thermo, Waltham, MA, USA) preceded reverse transcription (HiScript III RT SuperMix, Vazyme, Nanjing, China). A quantity of 500 ng total RNA was used for reverse transcription under standard conditions: 37 °C 15 min → 85 °C 5 s. Quantitative PCR (ABI QuantStudio 7 Pro, Thermo, Waltham, MA, USA) utilized automatic fluorescence threshold determination (QuantStudio v1.7, threshold = 10× baseline SD). The technical replicates maintained Ct variability <0.5 cycles. Differential gene expression vs. susceptible strains was calculated as 2^−ΔΔCt^ normalized to *rho*. Statistical significance was assessed via Student’s t-test by using GraphPad Prism 10 software (GraphPad, San Diego, CA, USA). Primers are listed in Table 1.

The whole-genome sequencing data were analyzed to investigate additional determinants of tigecycline resistance. The sequences of *ramA*, *ramR*, *rarA*, *acrR*, *acrA*, *acrB*, *soxS*, *oqxA*, *oqxB*, *tolC*, *rpsJ*, *ompK35*, and *ompK36* were compared with the reference genome *K. pneumoniae* MGH 78578 (GenBank accession no. CP000647) using BLAST+ 2.9.0 for mutation analysis. The *tet*(A) variant was characterized by aligning it with the *tet*(A) sequence in the *Escherichia coli* plasmid RP1 for *tet*(A) (GenBank accession no. X00006).

### 2.6. Efflux Pump Inhibition Test

Efflux-mediated tigecycline resistance was evaluated through Phe-Arg-β-naphthylamide (PAβN) modulation assays in two CRKP strains. Gradient PAβN concentrations (0/25/50/75 μg/mL) were co-incubated with strains during MIC determination via CLSI broth microdilution.

### 2.7. Bioinformatics Analysis

The CLC Workbench was utilized to identify antibiotic resistance genes, virulence genes, plasmid replicon types, and mutations via the Resfinder Database, Virulence Factor Database (VFDB), PlasmidFinder, and PointFinder. OriTfinder (https://bioinfo-mml.sjtu.edu.cn/oriTDB2/oriTfinder.php, assessed on 23 October 2024) was utilized to identify the mobility of plasmids. The sequence types (STs) were determined by Kleborate v2.0.4 (https://github.com/klebgenomics/Kleborate, accessed on 24 October 2024).

Core-genome SNPs from 62 CRKP strains (mapped to hvK2115, NZ_CP091326.1) were analyzed by maximum likelihood. The other three trees contained 30 ST11, 14 ST15, and 1 ST101 CRKP strains’ sequences obtained in this study, alongside 471 ST11, 249 ST15, and 71 ST101 strains from Institut Pasteur MLST databases. These sequences were constructed with RAxML followed by SNP calling and recombination removal with Snippy and Gubbins. All phylogenetic trees were visualized by iTOLs v7 (https://itol.embl.de/, accessed on 11 September 2024).

Hybrid-assembled plasmids were analyzed using NCBI BLAST+ v2.9.0 and BRIG. Bakta was used for fast annotation of complete chromosomes and plasmids. ISfinder and the Transposon Registry were utilized to perform comprehensive identification and annotation of mobile elements.

The Cochran-Armitage test for trend (C-A test) was used to examine the time trend of CRKP detection rates; *p*-values less than 0.05 were considered statistically significant.

### 2.8. Conjugation and Plasmid Analysis

Five MBL-CRKP donors and *Escherichia coli* strain EC600 (rifampin resistant) were cultured to reach the logarithmic phase (LB broth, 37 °C, 6 h). Donor and recipient bacterial solutions were prepared to a turbidity of 0.5 McFarland. Quantities of 400 µL of donor and 200 µL of recipient cultures were mixed and subsequently incubated at 37 °C for 18–24 h. Then, the mixture was inoculated on MacConkey agar plates containing rifampicin (2000 µg/mL) and IPM (4 µg/mL) at 37 °C for 24 h. Positive transconjugants were identified by MALDI-TOF-MS, antimicrobial susceptibility testing, and whole-genome sequencing.

## 3. Results

### 3.1. Prevalence and Clinical Characterization of Isolates

**Figure 1:**
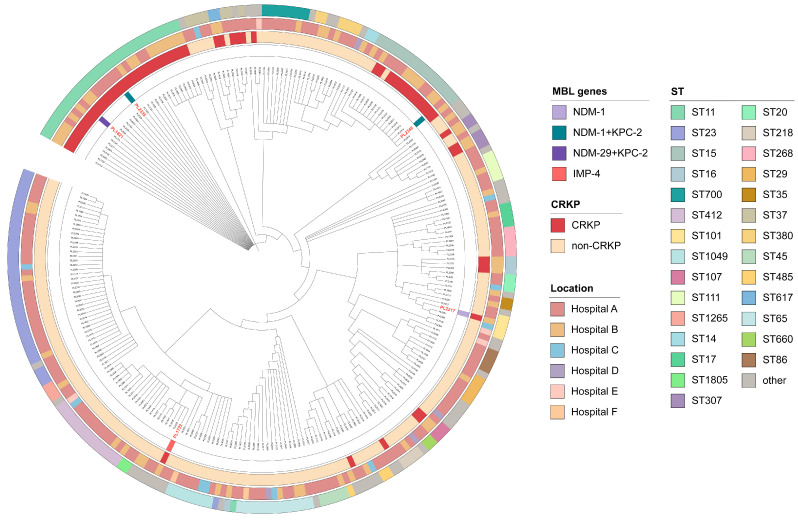
Phylogenetic trees of 255 *K. pneumoniae* strains. Red font indicates MBL-CRKP. From the inner circle to the outer circle, the first circle adjacent represents the presence or absence of the MBL genes, of which PL1821, PL2335, and PL2348 all carry two MBL genes. The second indicates the CRKP status, the third represents the region where the strains were isolated, and the fourth represents the sequence types of all strains. CRKP strains of the same ST type from different hospitals exhibit a clustering phenomenon.

Between 2016 and 2023, 255 clinical *K. pneumoniae* strains were isolated from five hospitals in Yangzhou City. Of the strains, 24% (62/255) were identified as CRKP. Within these 220 blood-derived strains, the ratio of CRKP was 23.18% (51/220). The majority of the CRKP strain carriers were adults, with 71% (44/62) being male and 84% (52/62) of the patients being over 50 years of age (Table S1). The percentage of CRKP increased with the passing of the year, from 7% in 2016 to 52% in 2022 (*p* < 0.05), and the number of CRKP isolates in 2022–2023 accounted for 45% (28/62) of all CRKP. Phylogenetic analysis revealed significant differences in clustering patterns between CRKP and non-CRKP. Most CRKP belonged to ST11 and ST15 and showed a clear tendency. In addition, non-CRKPs, predominantly ST23, also showed significant clustering. However, there were, but rarely, CRKP strains that appeared in clusters of non-CRKP strains (Figure 1).

In total, 8% (5/62) of CRKP strains carried MBL genes (Table S1). These MBL-CRKP strains were selected for further analysis. The five MBL-CRKP strains were obtained from blood samples collected at two hospitals. Two strains were from female patients, whereas three strains were from male patients, all of whom were over 50 years of age (Table S1). PL1821 and PL2335 belonged to ST11, whereas PL2348 belonged to ST15. For three of them, harboring two carbapenemase-encoding genes exhibited a high degree of genetic similarity not only to other strains within the same hospital but also to the same ST type CRKP strains from different hospitals and years with low numbers of differentiating SNPs (zero to four) (Figure 1). PL2217 and PL1722 carried one carbapenemase-encoding gene. PL2217 belonged to ST101, while PL1722 belonged to a rarely reported sequence type ST562.

The whole-genome SNP analysis of 501 ST11 strains revealed that 61 strains from Asia (including 30 strains in our study) formed a distinct clade with a clear clustering phenomenon. These strains were collected between 2017 and 2024, with most strains originating from humans and one from an animal. Except for one strain, all others harbored carbapenemase-encoding genes, primarily carrying *bla*_KPC-2_ (*n* = 59). Among these, six strains carried two carbapenemase-encoding genes, and one strain harbored *bla*_KPC-12_ (Figure S1). The whole-genome SNP analysis of 263 ST15 strains revealed that 14 strains from this study formed a distinct clade, which clustered with strains from Asia, Europe, and North America. The sample sources included humans (*n* = 32) and animals (*n* = 2). All the 14 ST15 strains in this study carried *bla*_KPC-2_, while most of the other strains in the cluster carried the *bla*_OXA-48_ gene (Figure S2). All 72 ST101 strains were collected between 2006 and 2022 from six different continents, with the majority originating from humans, while two strains isolated from the environment. PL2217 clustered with two strains, one isolated from Africa in 2009 (58986_ERR971974) and the other from Asia in 2015 (46634_HA2_7). PL2217 was the only strain carrying *bla*_NDM-1_, while 46634_HA2_7 carried *bla*_NDM-5_ (Figure S3).

### 3.2. Resistance Profiles and Associated Determinants

All 62 CRKP strains were MDR and resistant to at least five antibiotics. Notably, the five strains harboring MBLs displayed an even broader resistance profile, being resistant to at least 12 antibiotics. These MBL-CRKP strains consistently demonstrated resistance to CZA, carbapenems, other β-lactams, and CIP. Additionally, PL1722 and PL2348 exhibited reduced susceptibility to tigecycline (MIC = 8 and 4 μg/mL) (Table S2).

**Figure 2:**
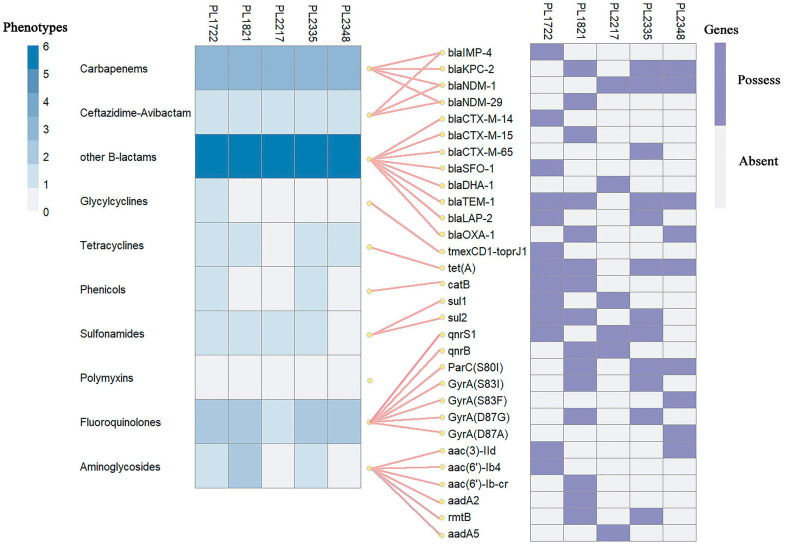
Resistance genes, resistance phenotypes, and the correspondence between them of the five strains. The left side shows the resistance phenotypes of strains; the color shades represent the number of antibiotics resistant to this antibiotic classes. The right side exhibits the resistance genes of strains; the purple square represents the existence of the resistance gene, while the gray one indicates the absence. The connecting lines in the middle represent the corresponding relationship between the resistance phenotypes and the resistance genes.

The MBL-CRKP strains exhibited good concordance between resistance phenotypes and genotypes (Figure 2). The carbapenem and CZA resistance phenotypes of these strains corresponded well with the class B metalloenzyme genes they harbored; all five strains carrying metalloenzyme-encoding genes showed high-level resistance to CZA (MIC ≥ 256 µg/mL).

### 3.3. Expression and Mutation Analysis of Tigecycline Resistance-Related Genes

Efflux pumps and locus mutations associated with tigecycline resistance were identified in two tigecycline non-susceptible strains (PL1722 and PL2348). Both strains harbored the *rpsJ* (V57L) mutation and type 1 *tet*(A). Additionally, truncation of the pore protein-coding genes *ompK35* and *ompK36*, glycine-aspartic acid duplication (GD), and the absence of *acrR* were observed on the chromosome of PL2348. PL1722, on the other hand, possessed a *tmexCD2-toprJ2* gene cluster.

**Figure 3:**
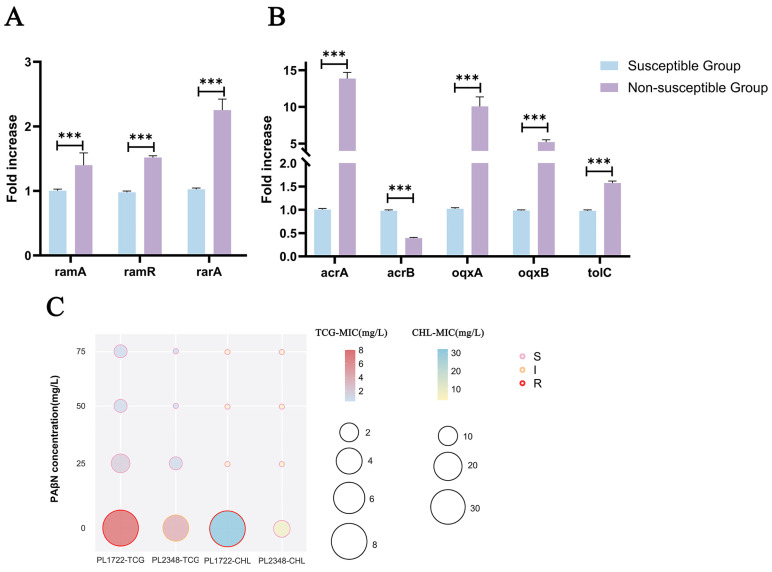
Average expression levels of tigecycline resistance-related genes. (**A**) Average expression levels of regulator genes ramA, ramR, rarA, and soxS. (**B**) Average expression levels of multidrug efflux pump-related genes acrA, acrB, oqxA, oqxB, and tolC. (**C**) Bubble plot illustrating the effects of PAβN concentrations on the MIC values of tigecycline (TCG) and chloramphenicol (CHL) in two tigecycline-nonsusceptibility strains. The *y*-axis represents PAβN concentrations (0, 25, 50, and 75 mg/L), while the *x*-axis lists the tested antibiotic. Bubble sizes correspond to the MIC values, with the color gradient indicating the MIC magnitude for each antibiotic (red for TCG and blue for CHL). The resistance categories (S, susceptible; I, intermediate; R, resistant) are indicated by bubble border colors: pink (S), orange (I), and red (R). Asterisks indicate statistically significant differences (*** indicate *p* < 0.001) in student *t*-tests.

In the tigecycline non-susceptible group, the efflux pump-encoding genes *acrA*, *oqxA*, and *oqxB* exhibited 5.5- to 14.7-fold higher expression compared with the tigecycline-susceptible strains (Figure 3B). Among them, PL2348 showed higher expression levels (3.0–6.7 fold) of *ramA*, *ramR*, *rarA*, and *soxS* genes (Figure 3A), while *oqxA*, *oqxB*, and *tolC* were more than ten-fold higher than those in the susceptible group. Unlike PL2348, PL1722 showed high *acrA* expression, which was 33.4-fold higher than that in the tigecycline-susceptible group (Figure 3B). Under the influence of PAβN, two tigecycline-nonsusceptible strains recovered sensitivity to tigecycline and chloramphenicol. Tigecycline exhibited a more pronounced decrease in MIC at pump inhibitor concentrations of 50 mg/L and 75 mg/L. The tigecycline MIC value of PL1722 decreased from 8 µg/mL to 0.5 µg/mL, and PL2348 decreased from 4 µg/mL to 1 µg/mL. The chloramphenicol MIC values of the two strains dropped below 4 μg/mL (Figure 3C).

### 3.4. Structural Analysis of bla_IMP-4_, tmexCD2-toprJ2 Coexistence Plasmid

**Figure 4:**
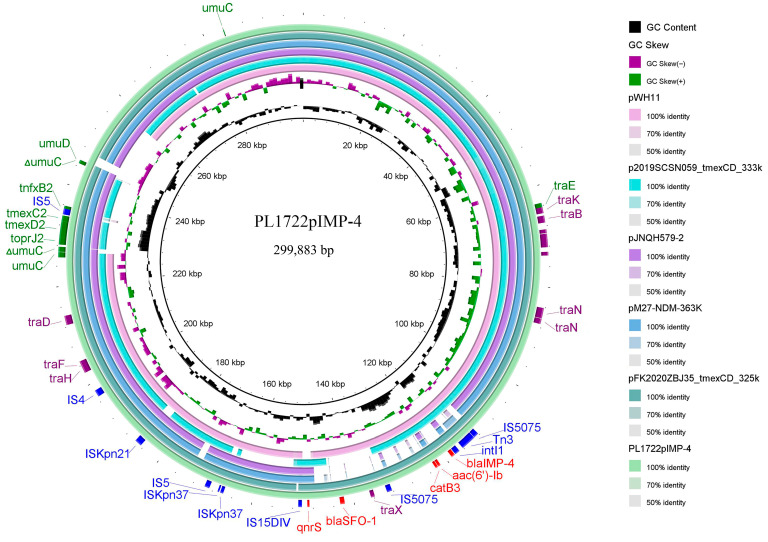
Comparative genome map of PL1722pIMP-4 with pWH11 (NZ_ON882017.1), p2019SCSN059_tmexCD_333k (NZ_ON169978.1), pJNQH579-2 (NZ_CP078148.1), pM27-NDM- 363K (CP130154.1), and pFK2020ZBJ35_tmexCD_325k (NZ_ON169979.1). The annotated genes on the outer cycle are the resistance genes (red), integrase genes (blue), and conjugation-related genes (purple) of PL1722pIMP-4, and the shades of the circles represent the sequence similarity between the five plasmids and PL1722pIMP-4s. PL1722pIMP-4 exhibits high similarity with the compared plasmids, sharing identical *bla*_IMP-4_ and *tmexCD2-toprJ2* genomic structures with some of them.

**Figure 5:**
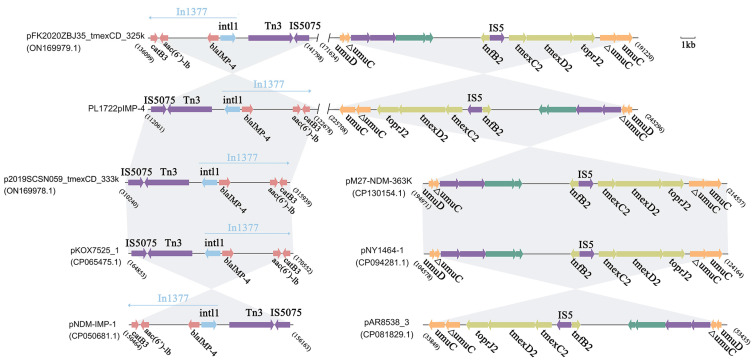
Linear comparison of the PL1722pIMP-4 *tmexCD2-toprJ2* gene cluster region with pFK2020ZBJ35_tmexCD_325k(ON169979.1), pM27-NDM-363K(CP130154.1), pNY1464-1(CP094281.1), and pAR8538_3(CP081829.1). Linear comparison of the PL1722pIMP-4 *bla*_IMP-4_-carrying integron region with pFK2020ZBJ35_tmexCD_325k(ON169979.1), p2019SCSN059_tmexCD_333k(ON169978.1), pKOX7525_1(CP065475.1), and pNDM-IMP-1(CP050681.1). Gray represents the sequence similarity.

The IncHI1B-type plasmid, PL1722pIMP-4, carried both *bla*_IMP-4_ and *tmexCD2-toprJ2*. PL1722pIMP-4 shared 99% identity and 88% coverage with plasmid pWH11 from a *K. pneumoniae* isolate from a human sample in Hubei Province, China. Notably, the *tmexCD2-toprJ2* gene cluster was absent in this plasmid. Meanwhile, PL1722pIMP-4 exhibited high similarity to four plasmids from different genera (>99% identity and >85% coverage) (Figure 4). The four plasmids possessed the same *tmexCD2-toprJ2* gene cluster structure as PL1722pIMP-4, forming a “*tnfxB2*-IS5-*tmexCD2*-*toprJ2*” cluster. This gene cluster was inserted into the *umuC* gene, with 6 bp direct repeats (ACCGCC) around the gene cluster. The linear analysis and comparison of the *tmexCD2-toprJ2* containing region was presented in Figure 4. Furthermore, *bla*_IMP-4_ was located in the class I integron named In*1377* and carried the *bla*_IMP-4_-*aac(6′)-Ib*-*catB3* gene cassette. The same integron structure was observed in the other four plasmids (Figure 5). Additionally, PL1722pIMP-4 contained *bla*_SFO-1_ and *qnrS* resistance genes, which might contribute to reduced susceptibility to β-lactamases and quinolone antibiotics.

### 3.5. Genomic Characterization and Comparative Genomic Analysis of Plasmids Carrying bla_NDM-1_ and bla_NDM-29_

**Figure 6:**
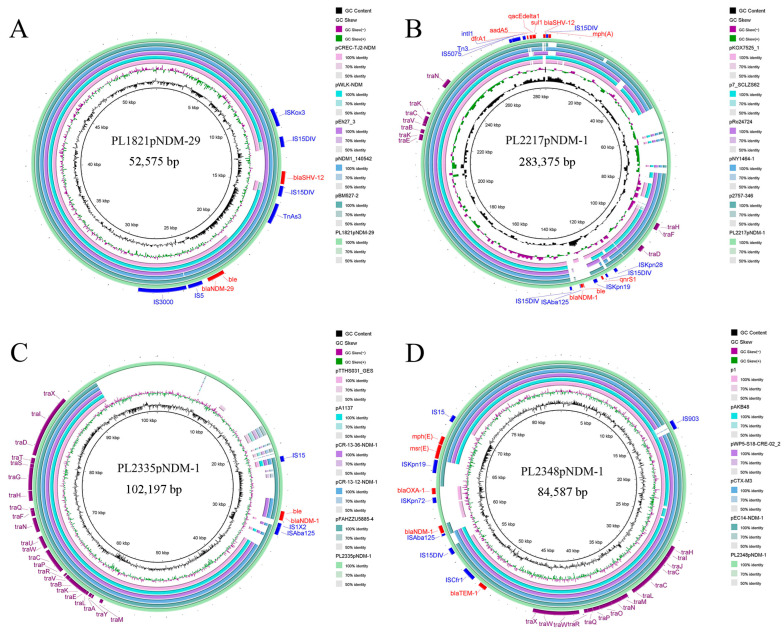
Comparative genome maps of *bla*_NDM_-carrying plasmids: the annotated genes on the outer cycle are the resistance genes (red), integrase genes (blue), and conjugation-related genes (purple), and the shades of the circles represent the sequence similarity between the four plasmids and the *bla*_NDM_-carrying plasmid. (**A**) Comparative genome map of PL1821pNDM-29 with pCREC-TJ2-NDM (NZ_KX960110.1), pWLK-NDM (NZ_CP038280.1), pEh27_3 (NZ_CP053694.1), pNDM1_140542 (CP103366.1), and pBM527-2 (NZ_CP041048.1). (**B**) Comparative genome map of PL2217pNDM-1 with pKOX7525_1 (NZ_CP065475.1), p7_SCLZS62 (CP082175.1), pRo24724 (NZ_CP021328.1), pNY1464-1 (NZ_CP094281.1), and p2757-346 (NZ_CP060810.1). (**C**) Comparative genome map of PL2335pNDM-1 with pTTHS031_GES (NZ_LC589514.1), pA1137 (NZ_MF190369.1), pCR-13-36-NDM-1 (NZ_MZ857202.1), pCR-13-12-NDM-1 (MN175388.1), and pFAHZZU5885-4 (NZ_CP135256.1). (**D**) Comparative genome map of PL2348pNDM-1 with p1 (NZ_CP129617.1), pAKB48(NZ_CP044337.1), pWP5-S18-CRE-02_2 (NZ_AP022128.1), pCTX-M3 (NC_004464.2), and pEC14-NDM-1(NZ_CP060926.1). The four *bla*_NDM_-carrying plasmids have distinct types. Except for PL2335pNDM-1, the remaining three plasmids share high similarity with compared plasmids. Among them, PL2217pNDM-1 and PL2348pNDM-1 both harbor MDR regions. Some compared plasmids share the same structure where the *bla*_NDM_ is located in the plasmids described in this study.

Four CRKP strains possessed *bla*_NDM_-carrying plasmids. BLAST analysis revealed that the four *bla*_NDM_-carrying plasmids shared high similarity with plasmids from different genera, with 97–100% identity and 83–99% coverage (Figure 6). All four plasmids had IS transposase insertions either upstream or downstream of *bla*_NDM_.

PL1821pNDM-29 was an IncX3 plasmid. *bla*_NDM-29_ was located downstream of IS5 and IS3000, sharing the same MBL gene-carrying structure with other plasmids. Simultaneously, this plasmid also carried the ESBL-encoding gene *bla*_SHV-12_ (Figure 6A). PL2217pNDM-1 was a 283,375 bp IncH1B plasmid. This plasmid contained two MDR regions. *ble*_MBL_ and *bla*_NDM-1_ were inserted into the backbone by ISAba125 transposases and ISKpn19 transposase, with *qnrS1* located between ISKpn19 transposase and IS15DIV. Meanwhile, another MDR region was located between 275,188 bp and 283,327 bp, which contained the *sul1* and *bla*_SHV-12_ resistance genes, as well as the *dfrA1* and *aadA5* located in the class I integron (Figure 6B). pL2348pNDM-1 was an IncM2 β-lactam resistance plasmid carrying three genes (*bla*_TEM-1_, *bla*_NDM-1_, and *bla*_OXA-1_). The region carrying *bla*_NDM-1_ and *bla*_OXA-1_ was inserted into the original backbone using two flanking ISAba125 and ISKpn19 transposases (Figure 6D). The PL2335pNDM-1 was an IncFII plasmid that only harbored one resistance gene *bla*_NDM-1_ and only shared 60–70% query coverage with the other five plasmids of different genera (Figure 6C).

### 3.6. Transferable CZA Resistance

Successful conjugation was observed with all three potential CRKP conjugative strains, resulting in transconjugants PL1722EC, PL2335EC, and PL2348EC. Sequencing of the transconjugants revealed that PL1722pIMP-4, PL2335pNDM-1, and PL2348pNDM-1 were successfully transferred to EC600, whereas PL2348pKPC-2 was not transferred to EC600. All the transconjugants exhibited a carbapenem-resistant phenotype and remained resistant to CZA. Compared with the original donors, two transconjugants maintained MICs of ≥8 µg/mL for all three carbapenems and high-level resistance to CZA (MIC > 256/4 µg/mL). PL1722pIMP-4 was transferred to EC600 with the help of conjugative plasmid PL1722pCTX. *bla*_IMP-4_ transfer conferred an ETP-resistant phenotype to EC600 (MIC = 2 μg/mL) and exhibited resistance to CZA (MIC = 256/4 μg/mL). Additionally, PL1722EC presented increased resistance to tigecycline with an MIC value of 4 µg/mL (Table S2).

## 4. Discussion

The emergence and transmission of MBL-CRKP pose a significant threat due to their heightened antibiotic resistance and associated challenges in clinical management [9]. This retrospective study comprehensively explored the epidemiological characteristics and phylogenetic relationships of CRKP strains from 2016 to 2023 in five major general hospitals of Yangzhou. Our research filled the gap in the study of the clinical epidemiological trend of CRKP strains in this region. Our findings highlighted the resistance of MBL-CRKP strains to carbapenems, CZA, and tigecycline, along with a detailed analysis of underlying resistance mechanisms and genomic transferability. These findings provide valuable insights into the current status and mechanisms of CRKP resistance in Yangzhou and underscore the critical importance of early and rapid diagnosis, precise treatment, and effective control measures to mitigate the spread of CRKP.

In recent years, the proportion of CRKP among *K. pneumoniae* isolates in Yangzhou has increased dramatically, rising by nearly nine-fold between 2016 and 2022. In our study, the CRKP proportion in blood samples was close to previous findings [24,25]. As a common CRE, CRKP in bloodstream infections is strongly associated with clinical treatment failure and high mortality rates [26,27,28,29]. Meanwhile, most strains were isolated in males aged over 50 years, which was consistent with the findings that age was an independent risk factor for CRKP infection and mortality in hospitalized patients [30,31]. Consistent with the prevalent ST in China, the predominant STs identified in our CRKP strains were ST11 and ST15 [32,33]. Moreover, the clustering of CRKP strains indicated potential hospital-wide and interhospital transmission [28]. The global phylogenetic analysis revealed a clustering phenomenon of ST11 strains, suggesting a region-specific spread in Asia. Both ST15 and ST101 strains exhibited clustering across multiple continents, indicating the potential for broader geographic dissemination. Furthermore, the majority of the strains carried carbapenem resistance genes, including different subtypes and cases where two resistance genes coexisted.

All the MBL-CRKP strains were identified across four distinct years (2017, 2018, 2022, and 2023), suggesting persistent endemic colonization in this region, necessitating proactive surveillance. What is worse, these strains exhibited multiple resistance phenotypes. In addition to resistance to first-line drug β-lactams, quinolones, and aminoglycosides, two strains exhibited reduced susceptibility to tigecycline, drastically limiting therapeutic options and amplifying public health risks. These strains harbored plasmids carrying *bla*_IMP-4_, *bla*_NDM-1_, and *bla*_NDM-29_, contributing to carbapenem and CZA resistance. The *bla*_IMP-4_ gene, along with *aac(6′)-Ib* and *catB3*, was located on an In1377-like class I integron. The presence of In1377 in the various plasmids suggested the crucial role of class I integrons in the dissemination of *bla*_IMP-4_ [34]. Furthermore, the coexistence of *bla*_IMP-4_ and *tmexCD2-toprJ2* harboring CRKP strain belonged to ST562, which is a common ST-carrying class I integron, highlights their potential as vectors for the spread of resistance genes [35].

We explored and identified several mechanisms that may lead to decreased susceptibility to tigecycline. First of all, we found elevated expression of *acrA* and *oqxAB* efflux pumps, which contributed to reduced tigecycline susceptibility [36,37]. Efflux pump inhibitors significantly lowered the MIC of tigecycline, highlighting the role of efflux pumps in drug resistance. Additionally, in our study, tigecycline-nonsusceptible CRKP strains harbored mutations in *rpsJ* and *tet*(A) variants and exhibited truncation of the outer membrane protein *ompK35* and glycine-aspartic acid duplication in *ompK36*, which were associated with increased tigecycline MIC in wild-type strains [38]. The cumulative impact of these mechanisms suggests that other potential efflux pumps and mutations may synergize with these mechanisms to collectively elevate antibiotic MICs.

Notably, we observed the coexistence of *bla*_IMP-4_ and the *tmexCD2-toprJ2* cluster in the same plasmid. The *tmexCD2-toprJ2* may further enhance tigecycline resistance. A potential regulatory molecule, *tnfxB2*, was located upstream of *tmexCD2-toprJ2* and may regulate the expression level of this efflux pump. Studies have shown that the T39R mutation in *tnfxB2* influenced the affinity of *tnfxB2* for DNA, leading to an increase in the MIC of tigecycline [18,39]. In addition, the *umuC* gene flanking *tnfxB2-tmexCD2-toprJ2* may be a “hotspot” for *tmexCD2-toprJ2* integration into IncHI1B plasmids [34,40]. Furthermore, we found that *tmexCD2-toprJ2* was present in plasmids from other *Enterobacteriaceae* strains and that these plasmid backbones had a high degree of similarity. At the same time, successful horizontal transfer of the IncHI1B plasmid carrying *bla*_IMP-4_ and *tmexCD2-toprJ2* significantly increased the tigecycline MIC in EC600, which demonstrated the important role of *tmexCD2-toprJ2* in tigecycline resistance. These suggested that the unique genetic structure of the plasmid may have been derived from sequence insertions in other genera and may lead to the spread of drug resistance. These findings also emphasized the need to monitor the resistance transfer across ecological niches from the perspective of “one health” in the future [41].

In the present study, *bla*_NDM_ was identified as the primary cause of CZA resistance and contributed to carbapenem resistance [42]. The coexistence of *bla*_NDM_ and *bla*_KPC-2_ was observed in the three strains. In recent years, the coexistence of *bla*_NDM-1_ and *bla*_KPC-2_ has been increasingly observed in most countries and regions [43,44,45,46]. What is worse, the successful transfer of *bla*_NDM-1_ harboring plasmids demonstrated acquired carbapenem and CZA resistance phenotypes. This was consistent with previous findings that *bla*_NDM-1_-carrying mobilizable plasmids made a great contribution to the emergence of KPC-2-NDM-1-CRKP [43]. The presence of mobilizable plasmids with MBL genes not only causes severe multidrug resistance but also poses significant challenges for clinical treatment and the whole ecosystem. Monitoring the transfer of CZA resistance in different ecological niches is crucial.

While this study provides robust mechanistic insights, it has limitations. Because of the targeted enrollment through a regional surveillance program, some clinical information was incomplete. In addition, the sampling strategy prioritized clinically urgent bloodstream infections, ensuring data reliability but potentially underrepresenting other infection types.

Therefore, in future research, it is necessary to increase the strength of drug resistance monitoring and explore more efficient and effective means of detecting high-risk drug resistance genes and efflux pumps. We should further construct a database of MBL-CRKP resistance genes and thoroughly explore the resistance mechanism and the evolutionary pathway of transmission. These may realize the precise combating, prevention, and control of drug resistance. Finally, the development of new clinical drugs should accelerate to cope with the increasingly severe drug resistance situation.

## 5. Conclusions

This study elucidates the current status of multi-drug resistance in MBL-CRKP, all of which demonstrate resistance to CZA. The transfer of the MBL gene-carrying plasmids can result in the acquisition of resistance to carbapenem and CZA. The coexistence of *tmexCD2-torpJ* and other efflux pumps with MBL genes has led to decreased susceptibility of MBL-CRKP strains to tigecycline. These severe phenomena of resistance coexistence and transfer emphasize the importance of monitoring and implementing effective control measures to prevent the spread of resistance across ecological niches.

## Figures and Tables

**Table 1:** Primers used in quantitative reverse transcriptase PCR.

Gene	Primer	Primer Sequence (5′→3′)
*ramA*	*ramA*-F	ACGATTTCCGCTCAGGTGATT
	*ramA*-R	CAATACGCAGCGGTTGATGC
*ramR*	*ramR*-F	CAGCTGGCACATTTCGTTGA
	*ramR*-R	GCTATATCGACTGGGGCGTG
*rarA*	*rarA*-F	TGCGGCCAGAAAATTTAGCG
	*rarA*-R	TTACCGCTGAGATCCGTTCG
*soxS*	*soxS*-F *soxS*-R	GCATCACGGTACGGAACATC AGTCGCCAGAAAGTCAGGATAC
*acrA*	*acrA*-F	TTTGTTCTGATGGCGCGTTG
	*acrA*-R	CCAGGTCACTGCTTCTCAGG
*acrB*	*acrB*-F	GATCATCGGCACCACGGTAT
	*acrB*-R	ACAGCGACGGGATAAACAGG
*oqxA*	*oqxA*-F	GGTGCTGGTGAAGTCGATCA
	*oqxA*-R	CAATGTATCCCGAGACGCGA
*oqxB*	*oqxB*-F	CAACTACGCCACGCTGAAAG
	*oqxB*-R	GGACGTTTTGCTCCTGCATC
*tolC*	*tolC*-F	TTTACCGCGCCAGGGTTATC
	*tolC*-R	CAGCAGGAAAATGCGCTCAG

## Data Availability

The GenBank accession numbers for plasmids PL1722pIMP-4, PL1821pNDM-29, PL2217pNDM-1, PL2335pNDM-1, and PL2348pNDM-1 are PQ279299, PQ279300, PQ279301, PQ279302, and PQ279303, respectively. The Bioproject accession number for 62 CRKP strains and three transconjugants is PRJNA1157991.

## Supplementary Materials

The following supporting information can be downloaded at: https://www.mdpi.com/article/10.3390/pathogens14030253/s1, Table S1: Characteristics of 62 CRKP strains. Table S2: Antimicrobial drug susceptibility profiles. Figure S1: Phylogenetic tree of 501 ST11 strains. Figure S2: Phylogenetic tree of 263 ST15 strains. Figure S3: Phylogenetic tree of 72 ST101 strains.
