# Supplementary material for: Emergence of Tigecycline-Nonsusceptible Carbapenem-Resistant Klebsiella pneumoniae with Metallo-β-Lactamase and Transferable Ceftazidime-Avibactam Resistance in China
Source: Pathogens. 2025 Mar 4;14(3):253. doi: 10.3390/pathogens14030253 (PMC11946248; doi:10.3390/pathogens14030253)
Supplement: Supplementary file 1 [file pathogens-14-00253-s001.zip › pathogens-3503798-supplementary.pdf]

Emergence of Tigecycline-Nonsusceptible Carbapenem-Resistant Klebsiella pneumoniae with Metallo-β-Lactamase and Transferable Ceftazidime-Avibactam Resistance in China

Yajuan Ni [1,2], Jiefu Peng [3], Yawen Xu [4], Liguo Zhu [3], Xiao Wang [5], Hui Jin [1,2,*], Huimin Qian [3,*]

Supplementary Materials

Table S1. Characteristics of 62 CRKP strains.

Isolate	Isolation source	Isolation year	Gender	Age	Carbapenemases	Plasmids carrying MBL genes
PL1608	blood	2016	female	60	-	/
PL1701	blood	2017	male	63	-	/
PL1712	blood	2017	male	69	KPC-2	/
PL1721	blood	2017	male	76	KPC-2	/
PL1722	blood	2017	female	54	IMP-4	PL1722pIMP-4
PL1723	blood	2017	male	64	KPC-2	/
PL1724	blood	2017	male	64	KPC-2	/
PL1725	blood	2017	male	63	-	/
PL1726	blood	2017	female	47	-	/
PL1806	blood	2018	male	46	KPC-2	/
PL1809	blood	2018	male	67	-	/
PL1814	cerebrospinal fluid	2018	male	37	KPC-2	/
PL1816	blood	2018	female	72	KPC-2	/
PL1821	blood	2018	female	75	KPC-2; NDM-29	PL1821pNDM-29
PL1823	blood	2018	male	88	KPC-2	/
PL1826	blood	2018	male	71	KPC-2	/
PL1926	blood	2019	female	69	KPC-2	/
PL1929	blood	2019	female	67	KPC-2	/
PL1930	puncture fluid	2019	male	56	KPC-2	/
PL2001	blood	2020	male	32	KPC-2	/
PL2002	blood	2020	male	55	KPC-2	/
PL2003	blood	2020	male	65	KPC-2	/
PL2004	blood	2020	female	66	KPC-2	/
PL2005	sputum	2020	male	51	KPC-2	/
PL2006	urine	2020	male	57	KPC-2	/
PL2009	drainage	2020	female	62	KPC-2	/
PL2017	blood	2020	male	50	KPC-2	/
PL2039	blood	2020	male	73	KPC-2	/
PL2042	blood	2020	male	71	KPC-2	/
PL2102	urine	2021	male	79	KPC-2	/
PL2103	blood	2021	male	36	KPC-2	/
PL2104	blood	2021	female	57	KPC-2	/
PL2106	blood	2021	male	59	KPC-2	/
PL2118	sputum	2021	male	75	KPC-2	/
PL2201	blood	2022	male	62	KPC-2	/
PL2203	blood	2022	female	54	KPC-2	/
PL2204	blood	2022	female	65	KPC-2	/
PL2205	cerebrospinal fluid	2022	female	65	KPC-2	/
PL2207	blood	2022	female	44	-	/
PL2212	blood	2022	male	58	KPC-2	/
PL2213	blood	2022	male	81	KPC-2	/

Table S1 Characteristics of 62 CRKP strains (continued)

Isolate	Isolation source	Isolation year	Gender	Age	Carbapenemases	Plasmids carrying MBL genes
PL2214	blood	2022	female	54	KPC-2	/
PL2215	blood	2022	male	66	KPC-2	/
PL2216	blood	2022	female	54	KPC-2	/
PL2217	blood	2022	male	59	NDM-1	PL2217pNDM-1
PL2219	blood	2022	male	82	KPC-2	/
PL2221	blood	2022	male	39	KPC-2	/
PL2223	blood	2022	male	67	-	/
PL2227	blood	2022	male	44	KPC-2	/
PL2301	cerebrospinal fluid	2023	female	47	KPC-2	/
PL2313	blood	2023	female	83	KPC-2	/
PL2314	blood	2023	male	93	KPC-2	/
PL2320	blood	2023	male	54	KPC-2	/
PL2324	blood	2023	male	54	KPC-2	/
PL2326	blood	2023	male	75	KPC-2	/
PL2335	blood	2023	male	58	KPC-2; NDM-1	PL2335pNDM-1
PL2348	blood	2023	male	73	KPC-2; NDM-1	PL2348pNDM-1
PL2353	blood	2023	male	85	KPC-2	/
PL2370	blood	2023	male	67	OXA-48	/
PL2371	sputum	2023	male	69	OXA-48	/
PL2372	sputum	2023	male	36	OXA-48	/
PL2373	blood	2023	male	77	KPC-2	/

-: no carbapenemases were detected; /: no plasmids carrying MBL genes were detected

Table S2. Antimicrobial drug susceptibility profiles.

Antibiotics	MIC (μg/mL)/antimicrobial susceptibility							
			Strains			Transconjugants		
	PL1722	PL1821	PL2217	PL2335	PL2348	PL1722EC	PL2335EC	PL2348EC
ETP	8/R	> 8/R	> 8/R	> 8/R	> 8/R	2/R	> 8/R	> 8/R
MEM	4/R	> 64/R	64/R	> 64/R	> 64/R	1/S	32/R	16/R
IPM	4/R	> 64/R	16/R	> 64/R	> 64/R	2/I	32/R	16/R
CZA	256/4/R	> 256/4/R	> 256/4/R	> 256/4/R	> 256/4/R	256/4/R	> 256/4/R	> 256/4/R
TCG	8/R	0.5/S	0.5/S	2/S	4/I	4/I	≤ 0.25/S	≤ 0.25/S
TET	> 16/R	> 16/R	2/S	> 16/R	> 16/R	> 16/R	2/S	≤ 1/S
CHL	32/R	≤ 4/S	≤ 4/S	16/R	8/S	≤ 4/S	≤ 4/S	≤ 4/S
SXT	> 8/152/R	> 8/152/R	> 8/152/R	> 8/152/R	≤ 0.5/9.5/S	≤ 0.5/9.5/S	≤ 0.5/9.5/S	≤ 0.5/9.5/S
CST	0.5/I	0.5/I	0.5/I	0.5/I	0.5/I	≤ 25/S	0.5/I	≤ 0.25 /I
CTX	> 16/R	> 16/R	> 16/R	> 16/R	> 16/R	> 16/R	> 16/R	> 16/R
CAZ	> 16/R	> 16/R	> 16/R	> 16/R	> 16/R	> 16/R	> 16/R	> 16/R
AMP	> 32/R	> 32/R	> 32/R	> 32/R	> 32/R	> 32/R	> 32/R	> 32/R
SAM	> 32/16/R	> 32/16/R	> 32/16/R	> 32/16/R	> 32/16/R	> 32/16/R	> 32/16/R	> 32/16/R
ATM	> 16/R	> 16/R	> 16/R	> 16/R	> 16/R	16/R	≤ 0.25/S	≤ 0.25/S
FOX	> 64/R	> 64/R	> 64/R	> 64/R	> 64/R	> 64/R	> 64/R	> 64/R
CIP	> 2/R	> 2/R	1/R	> 2/R	> 2/R	> 2/R	0.25/S	0.125/S
NAL	32/R	> 32/R	≤ 4/S	> 32/R	> 32/R	> 32/R	> 32/R	> 32/R
AMK	≤ 4/S	> 64/R	≤ 4/S	> 64/R	≤ 4/S	≤ 4/S	≤ 4/S	≤ 4/S
AZM	＞ 64/R	＞ 64/R	＞ 64/R	＞ 64/R	＞ 64/R	32/R	≤ 2/S	64/R
STR	> 32/R	> 32/R	8/S	≤ 4/S	≤ 4/S	> 32/R	8/S	8/S
NIT	128/R	256/R	64/I	256/R	256/R	≤ 32/R	≤ 32/R	≤ 32/R

ETP, ertapenem; MEM, meropenem; IPM, imipenem; CZA, Ceftazidime-Avibactam; TGC, tigecycline; TET, tetracycline; CHL, chloramphenicol; SXT, trimethoprim-sulfamethoxazole; CST, colistin; CTX, ceftriaxone; CAZ, ceftazidime; AMP, ampicillin; SAM, ampicillin/sulbactam; ATM, aztreonam; FOX, cefoxitin; CIP, ciprofloxacin; NAL, nalidixic acid; AMK, amikacin; AZM, azithromycin; STR, streptomycin; NIT, nitrofurantoin

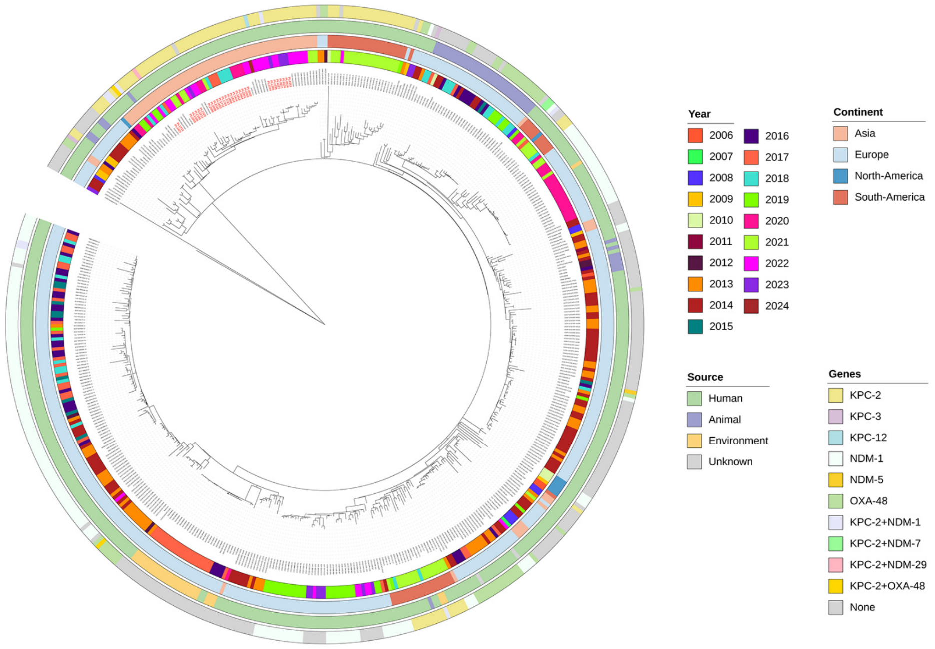

Figure S1. Phylogenetic tree of 501 ST11 strains. The inner to outer circles represent the year of isolation, geographic region, strain source and the carbapenem resistance genes carried by the strains. Strains isolated in this study are highlighted in red for emphasis.

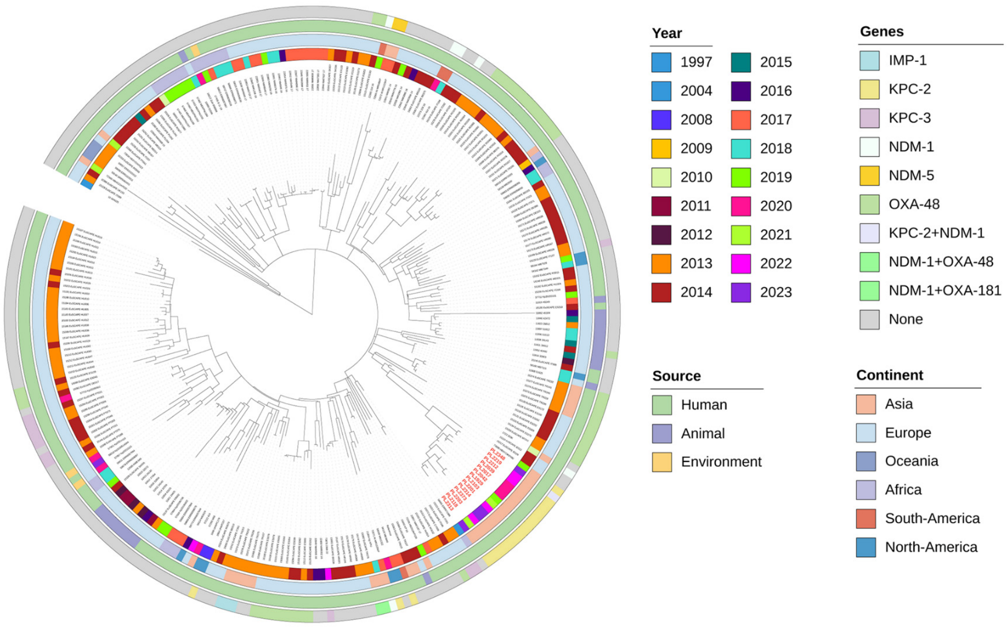

Figure S2. Phylogenetic tree of 263 ST15 strains. The inner to outer circles represent the year of isolation, geographic region, strain source and the carbapenem resistance genes carried by the strains. Strains isolated in this study are highlighted in red for emphasis.

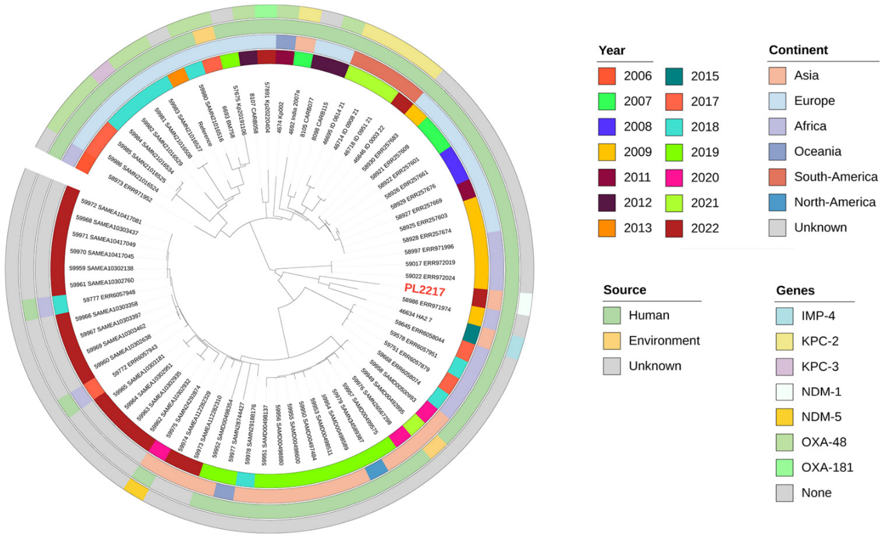

Figure S3. Phylogenetic tree of 72 ST101 strains. The inner to outer circles represent the year of isolation, geographic region, strain source and the carbapenem resistance genes carried by the strains. Strains isolated in this study are highlighted in red for emphasis.
